# Upadacitinib in a Refractory Case of Alpha-1 Antitrypsin Deficiency-Related Panniculitis

**DOI:** 10.7759/cureus.92467

**Published:** 2025-09-16

**Authors:** Majda Chaoui, Axel De Greef, Liliane Marot, Marie Baeck

**Affiliations:** 1 Dermatology, Cliniques Universitaires Saint-Luc, Brussels, BEL; 2 Institute of Experimental and Clinical Research (IREC) Pneumology, ENT and Dermatology Pole (LUNS), Université Catholique de Louvain, Brussels, BEL; 3 Anatomopathology, Cliniques Universitaires Saint-Luc, Brussels, BEL

**Keywords:** alpha-1 antitrypsin deficiency, jak inhibitors, panniculitis, pisz phenotype, upadacitinib

## Abstract

Alpha-1 antitrypsin (AAT) deficiency is a rare inherited disorder that can lead to emphysema, liver cirrhosis, or, more rarely, panniculitis. Treatment for AAT deficiency-associated panniculitis is limited and often unsatisfactory. We report the case of a 62-year-old man with recurrent, painful panniculitis lesions on the thighs and hips, associated with fever and weight loss. Laboratory tests revealed low serum AAT levels (0.56 g/L) and elevated C-reactive protein (11.3 mg/L). PiSZ phenotyping confirmed the diagnosis of AAT deficiency. Standard treatments were ineffective or contraindicated. Upadacitinib, a JAK1 inhibitor, was initiated at 45 mg/day for three months, leading to a substantial reduction in flare frequency and pain, with lesions healing more rapidly, and a sustained clinical benefit persisting for three months after discontinuation. This is the first reported case of AAT deficiency-associated panniculitis treated with upadacitinib. Although full remission was not achieved, symptom improvement and lack of adverse events suggest a potential role for JAK inhibitors in such cases. Upadacitinib may represent a novel therapeutic approach for AAT deficiency-related panniculitis.

## Introduction

Alpha-1 antitrypsin (AAT) deficiency is a rare and often under-recognized inherited disorder caused by autosomal co-dominant mutations in the SERPINA1 gene (OMIM*107400). It is typically characterized by emphysema, liver cirrhosis, and, more rarely, panniculitis, which affects approximately one in 1000 individuals with severe AAT deficiency, mainly in predominantly white populations [1-3]. Panniculitis has been reported to occur in various genotypes, including ZZ (62-70% of cases), MZ, SZ, SS, and MS, and is thought to result from an unopposed proteolysis in the skin, manifesting as painful, erythematous, sometimes ulcerated, nodules or plaques on the thighs or buttocks [4-6]. Current therapeutic options for AAT deficiency-related panniculitis often yield mitigated results (dapsone, doxycycline) or are difficult to access (intravenous infusion of AAT) [6,7]. Interestingly, AAT augmentation therapy has been shown to downregulate cytokines involved in the Janus kinase-signal transducer and activator of transcription (JAK-STAT) pathway [8]. In parallel, JAK inhibitors have demonstrated efficacy in certain connective tissue disease-associated panniculitis [9-11], suggesting that targeting this pathway could represent a promising therapeutic approach for AAT deficiency-related panniculitis.

## Case presentation

**Figure 1:**
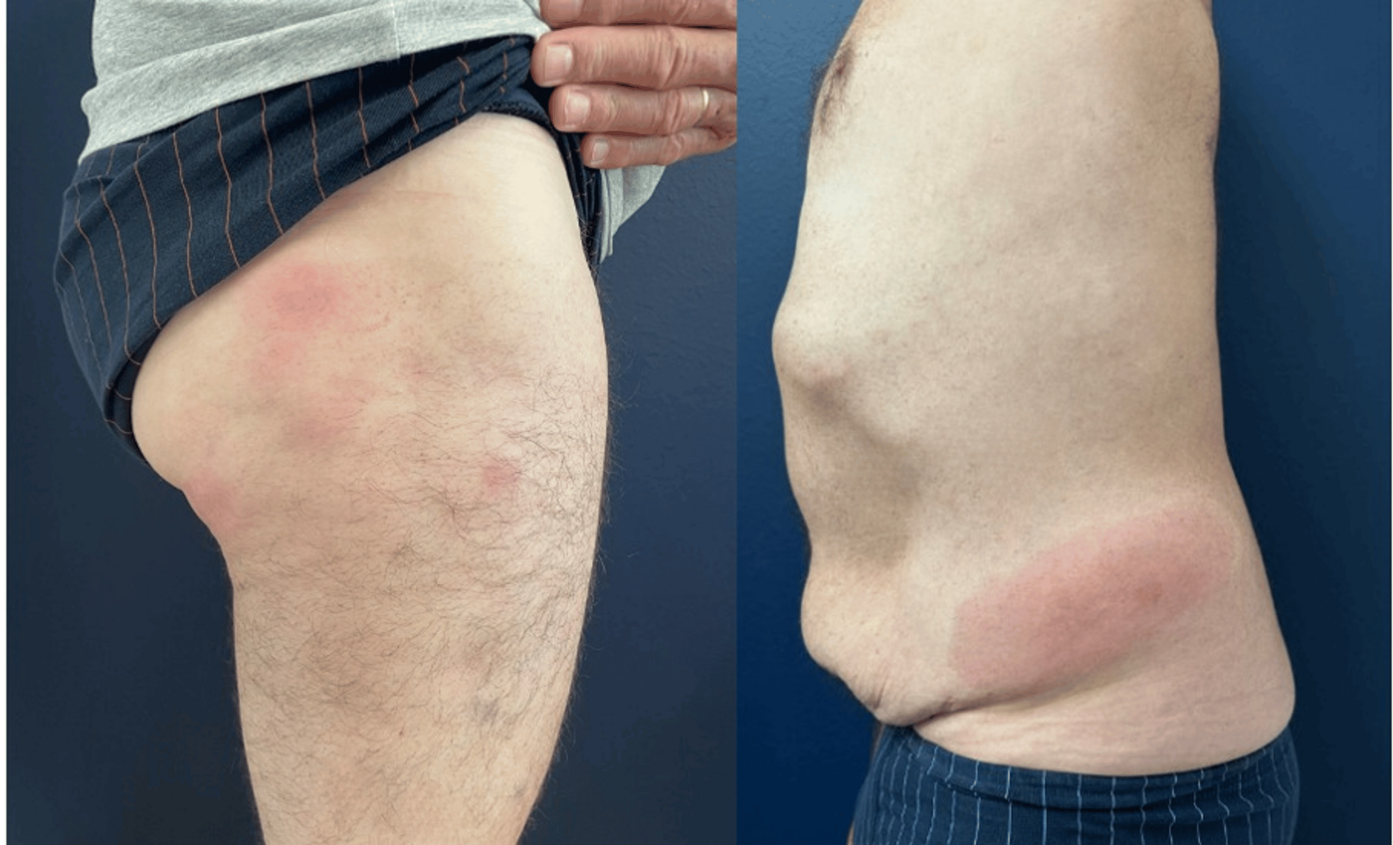
Clinical features of AAT deficiency-related panniculitis AAT: alpha-1 antitrypsin

We report the case of a 62-year-old male patient who presented with recurrent, painful dermo-hypodermal nodules in various locations, mainly on the thighs and hips. These lesions appeared approximately every two weeks, resolved spontaneously within 10 to 14 days, and healed without scarring (Figure 1). The flares were associated with systemic symptoms, including a low-grade fever and a significant weight loss of 15 kg over several months. The patient had a stable asthmatic form of chronic obstructive pulmonary disease and hypertension.

**Figure 2:**
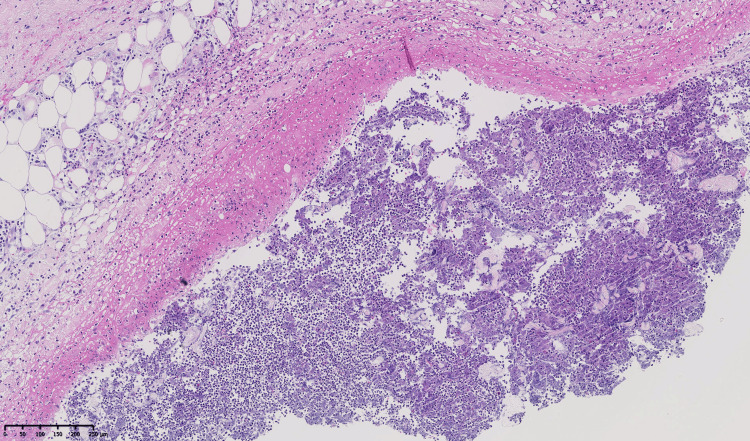
Biopsy of the thigh The biopsy shows dense neutrophilic infiltration with abscess formation in the subcutaneous tissue (hematoxylin and eosin, ×100). Microscopic examination shows a dermis with a mild perivascular lymphocytic infiltrate and a subcutis with a polymorphous septal and lobular inflammatory infiltrate. In the deeper fat, a collection of degenerated neutrophils is bordered by a necrotic eosinophilic wall. No granulomas or lymphomatous proliferation are identified. PAS, Gram, and Alcian blue stains reveal no pathogens.

Histopathology of a deep skin biopsy showed panniculitis with subcutaneous abscess formation consistent with necrotizing neutrophilic panniculitis (Figure 2). Laboratory tests were unremarkable, except for a low serum AAT level of 0.56 g/L (normal values: 0.85-1.94 g/L) and a glucose-6-phosphate dehydrogenase (G6PD) deficiency of 4 IU/g of hemoglobin (normal values: 6.1-15.2 IU/g Hb) (Table 1).

**Table 1:** Laboratory findings L: data are abnormally low, H: data are abnormally high, AAT: alpha-1 antitrypsin, G6PD: glucose-6-phosphate dehydrogenase

Parameter	Unit	Normal values
Hemoglobin	13.4	13.3-16.7 g/dL
Hematocrit	40	38-48%
Red blood cell count	4.33	4.00-6.00 10⁶/µL
White blood cell count	5.01	4.00-10.00 10³/µL
Platelet count	269	150-450 10³/µL
C-reactive protein	11.3 (H)	≤5.0 mg/L
Creatinine	0.84	0.60-1.30 mg/dL
Glomerular filtration rate	95	≥60 mL/min/1.73m²
Sodium	138	135-145 mmol/L
Potassium	4.60	3.50-5.00 mmol/L
Calcium total	2.34	2.20-2.55 mmol/L
Phosphorus	1.00	0.81-1.45 mmol/L
Aspartate aminotransferase	21	19-48 U/L
Alanine aminotransferase	21	10-40 U/L
QuantiFERON	Negative, latent tuberculosis or recent infection with *Mycobacterium tuberculosis* is unlikely	
Lipase	18	13-60 UI/L
AAT	0.56 (L)	0.85-1.94 g/L
G6PD	4.1 (L)	6.1-15.2 UI/g Hb

Main differential diagnoses included erythema nodosum, lupus panniculitis, infectious panniculitis, pancreatic panniculitis, and erythema induratum [12]. However, the biopsy demonstrated a lobular and septal neutrophilic panniculitis with subcutaneous abscesses and no vasculitis, which does not fit erythema nodosum (predominantly septal panniculitis without vasculitis) and lacks the lymphocytic predominance, hyaline fat necrosis, mucin deposition, and interface changes of lupus panniculitis. Pancreatic panniculitis was unlikely given a normal lipase (18 U/L), and erythema induratum/tuberculosis-related panniculitis was less probable with a negative QuantiFERON and absence of vasculitis. There was no granulomatous inflammation or lymphomatous proliferation, and special stains revealed no organisms. In the setting of low serum AAT (0.56 g/L) and a PiSZ phenotype, these findings favored AAT deficiency-related panniculitis. Subsequent treatments with hydroxychloroquine 200 mg daily (three months), colchicine 1 mg daily (three months), and doxycycline 200 mg daily (one month) were all unsuccessful, and dapsone was contraindicated due to the patient’s G6PD deficiency. Although intravenous augmentation therapy with plasma-purified AAT is the most effective treatment option [5-8], it was not available in Belgium.

**Figure 3:**
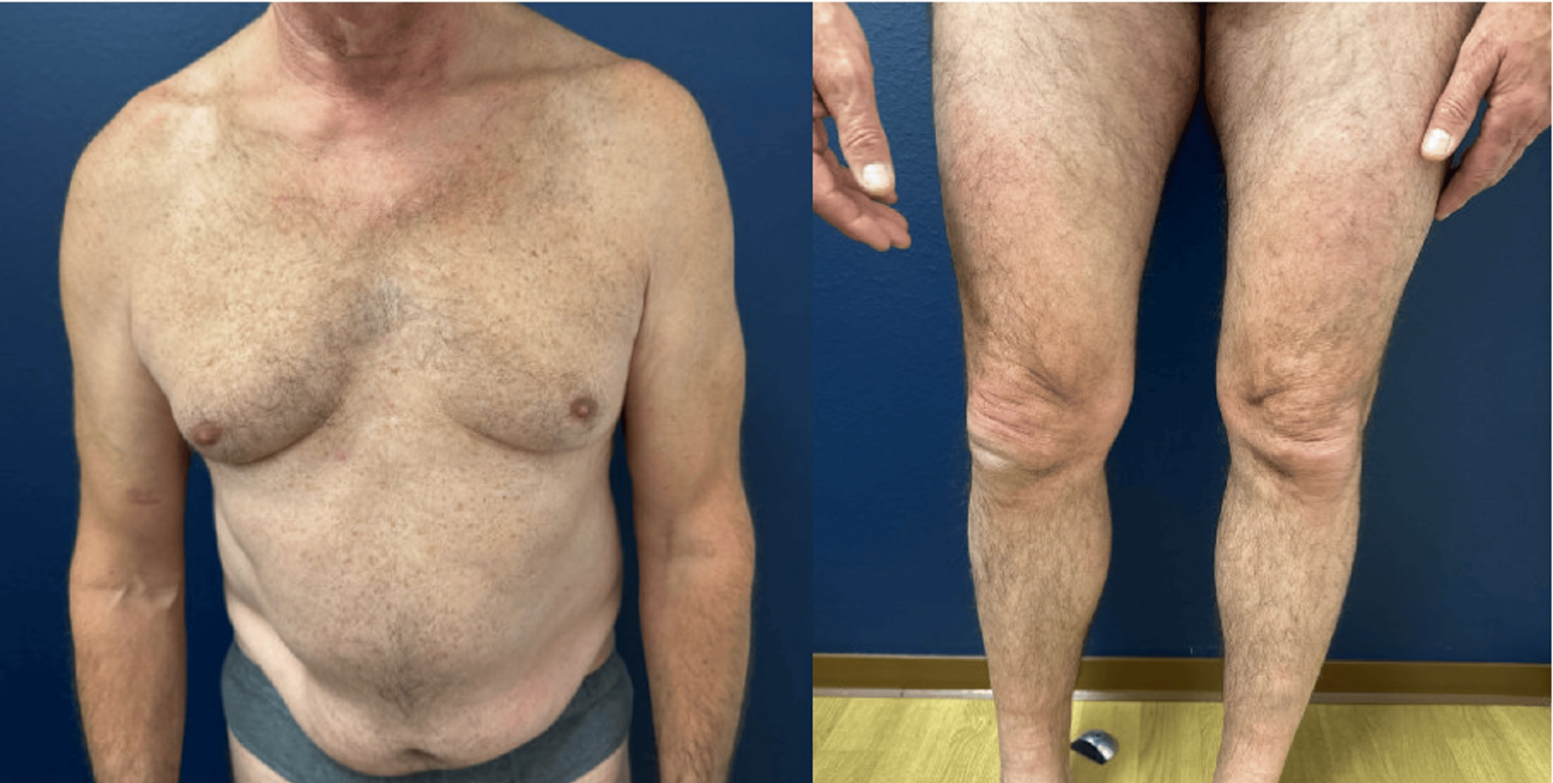
Clinical improvement after three months of upadacitinib treatment

The patient was subsequently treated with upadacitinib (a selective JAK1 inhibitor) at 45 mg/day for three months. Despite an incomplete response, the treatment resulted in a substantial reduction in the frequency and severity of flares (Figure 3), and no adverse events were reported. Upadacitinib was then discontinued for three months. During this period, the patient reported that the improvement persisted, with fewer and less painful episodes than before starting treatment.

## Discussion

While panniculitis is a much rarer complication of AAT deficiency compared to pulmonary and hepatic involvement, it can present as an initial symptom and significantly impair a patient's quality of life. To date, its treatment remains poorly defined, largely due to the rarity of the condition [13].

JAK inhibitors have significantly improved the management of several immune-mediated dermatologic diseases, including atopic dermatitis, vitiligo, and alopecia areata. Beyond their classical indications, JAK inhibitors have also shown effectiveness in granulomatous skin diseases [14,15] and neutrophilic dermatoses such as pyoderma gangrenosum [9] or Sweet syndrome [16].

Our therapeutic choice was based not only on reports of JAK inhibitor effectiveness in other neutrophilic or connective tissue-related panniculitis, such as lupus erythematosus panniculitis [10], rheumatoid neutrophilic panniculitis [9], or dermatomyositis-associated panniculitis [11], but also on mechanistic evidence implicating the JAK-STAT pathway in neutrophil-driven inflammation. Indeed, IFN-γ stimulation has been shown to activate JAK1/JAK2 with downstream STAT1 phosphorylation in human neutrophils [17], an effect that can be inhibited by JAK inhibitors. These findings provide a biological explanation for the potential benefit of JAK-STAT blockade in neutrophil-rich panniculitis (like in our patient) and, in the absence of other effective options, support the exploratory use of upadacitinib in our case of AAT deficiency-related panniculitis. Additionally, in our setting, the choice of upadacitinib over other JAK inhibitors previously reported in panniculitis was also dictated by restricted off-label access in Belgium and the practical availability of upadacitinib.

While complete remission was not achieved, the patient's quality of life improved, suggesting a potential therapeutic role for JAK1 inhibition in this context. A limitation of our report is the absence of a standardized tool to quantify lesion severity and response, as no validated scoring system exists for AAT deficiency-related panniculitis. Whether a longer treatment duration would have provided additional benefit remains unknown, as no data are currently available to inform duration-response relationships for JAK inhibition in this condition.

## Conclusions

This case contributes to the limited existing literature on the use of JAK inhibitors in AAT deficiency-related panniculitis and suggests that upadacitinib may be a potential therapeutic option in selected cases. However, further prospective and comparative studies, and on larger cohorts, are needed to better define its role in the management of this rare and often debilitating extrapulmonary manifestation of AAT deficiency.
